# Supplementary material for: Impact of interhospital competition on mortality of patients operated on for colorectal cancer faced to hospital volume and rurality: A cross-sectional study
Source: PLoS One. 2024 Jan 25;19(1):e0291672. doi: 10.1371/journal.pone.0291672 (PMC10810549; doi:10.1371/journal.pone.0291672)
Supplement: S1 Appendix — (DOCX) [file pone.0291672.s001.docx]

##### **Appendix 3.** *CHARLSON COMORBITY SCORE CALCULATION*

| ***VARIABLE*** | **Coef** | **ICD-10 CODES** |
| --- | --- | --- |
| **Congestive heart failure** | 1 | I110, I130, I132, I50, I500, I501, I509 |
| **Myocardial infarction** | 1 | I21, I2,2 I252, I255 |
| **Peripheral vascular disease** | 1 | I70, I700, I701, I702, I708, I709, I71, I710, I711, I712, I713, I714, I715, I716, I718, I719, I731, I738, I739, I771, I790, I792, K551, K558, K559, Z958, Z959 |
| **Cerebrovascular disease** | 1 | G45, G450, G451, G452, G453, G454, G458, G459, G46, G460, G461, G462, G463, G464, G465, G466, G467, G468, H340, I60, I600, I601, I602, I603, I604, I605, I606, I607, I608, I609, I61, I610, I611, I612, I613, I614, I615, I616, I618, I619, I62I, 620, I621, I629, I63, I630, I631, I632, I633, I634, I635, I636, I638, I639, I64, I65, I650, I651, I652, I653, I658, I659, I66, I660, I661, I662, I663, I664, I668, I669, I67, I670, I671, I672, I673, I674, I675, I676, I677, I678, I679, I68, I680, I681, I682, I688, I69,  I690, I691, I692, I693, I694, I698 |
| **Dementia** | 1 | F00, F000, F0000, F00000, F00001, F00002, F0001, F00010, F00011, F00012, F0002, F00020, F00021, F00022, F0003, F00030, F00031, F00032, F0004, F00040, F00041, F00042, F001, F0010, F00100, F00101, F00102, F0011, F00110, F00111, F00112, F0012, F00120, F00121, F00122, F0013, F00130, F00131, F00132, F0014, F00140, F00141, F00142, F002, F0020, F00200, F00201, F00202, F0021, F00210, F00211, F00212, F0022, F00220, F00221, F00222, F0023, F00230, F00231, F00232, F0024, F00240, F00241, F00242, F009, F0090, F00900, F00901, F00902, F0091, F00910, F00911, F00912, F0092, F00920, F00921, F00922, F0093, F00930, F00931, F00932, F0094, F00940, F00941, F00942, F01, F010, F0100, F01000, F01001, F01002, F0101, F01010, F01011, F01012, F0102, F01020, F01021, F01022, F0103, F01030, F01031, F01032, F0104, F01040, F01041, F01042, F011, F0110, F01100, F01101, F01102, F0111, F01110, F01111, F01112, F0112, F01120, F01121, F01122, F0113, F01130, F01131, F01132, F0114, F01140, F01141, F01142, F012, F0120, F01200, F01201, F01202, F0121, F01210, F01211, F01212, F0122, F01220, F01221, F01222, F0123, F01230, F01231, F01232, F0124, F01240, F01241, F01242, F013, F0130, F01300, F01301, F01302, F0131, F01310, F01311, F01312, F0132, F01320,  F01321, F01322, F0133, F01330, F01331, F01332, F0134, F01340, F01341, F01342, F018, F0180, F01800, F01801, F01802, F0181, F01810, F01811, F01812, F0182, F01820, F01821, F01822, F0183, F01830, F01831, F01832, F0184, F01840, F01841, F01842, F019, F0190, F01900, F01901, F01902, F0191, F01910, F01911, F01912, F0192, F01920, F01921, F01922, F0193, F01930, F01931, F01932, F0194, F01940, F01941, F01942, F02, F020, F0200, F02000, F02001, F02002, F0201, F02010, F02011, F02012, F0202, F02020, F02021, F02022, F0203, F02030, F02031, F02032, F0204, F02040, F02041, F02042, F021, F0210, F02100, F02101, F02102, F0211, F02110, F02111, F02112, F0212, F02120, F02121, F02122, F0213, F02130, F02131, F02132, F0214, F02140, F02141, F02142, F022, F0220, F02200, F02201, F02202,  F0221, F02210, F02211, F02212, F0222, F02220, F02221, F02222, F0223, F02230, F02231, F02232, F0224, F02240, F02241, F02242, F023, F0230, F02300, F02301, F02302, F0231, F02310, F02311, F02312, F0232, F02320, F02321, F02322, F0233, F02330, F02331, F02332, F0234, F02340, F02341, F02342, F024, F0240, F02400, F02401, F02402, F0241, F02410, F02411, F02412, F0242, F02420, F02421, F02422, F0243, F02430, F02431, F02432, F0244, F02440, F02441, F02442, F028, F0280,  F02800, F02801, F02802, F0281, F02810, F02811, F02812, F0282, F02820, F02821, F02822, F0283, F02830, F02831, F02832, F0284, F02840, F02841, F02842, F03, F03+0, F03+00, F03+01, F03+02, F03+1, F03+10, F03+11, F03+12, F03+2, F03+20, F03+21, F03+22, F03+3, F03+30, F03+31, F03+32, F03+4, F03+40, F03+41, F03+42, F051, G30, G300, G301, G308, G309, G311 |
| **Chronic pulmonary disease** | 1 | ( I278, I279, J40, J41, J410, J411, J418, J42, J43, J430, J431, J432, J438, J439, J44, J440, J441, J448, J449, J45, J450, J451, J458, J459, J46, J47, J60, J61, J62, J620, J628, J63, J630, J631, J632, J633, J634, J635, J638,  J64, J65, J66, J660, J661, J662, J668, J67, J670, J671, J672, J673, J674, J675, J676, J677, J678, J679, J684, J701, J703 |
| **Connective tissue disease** | 1 | M05, M050, M0500, M0501, M0502, M0503, M0504, M0505, M0506, M0507, M0508, M0509, M051, M0510, M0511, M0512, M0513, M0514, M0515, M0516, M0517, M0518, M0519, M052, M0520, M0521, M0522, M0523, M0524, M0525, M0526, M0527, M0528, M0529, M053, M0530, M0531, M0532, M0533, M0534, M0535, M0536, M0537, M0538, M0539, M058, M0580, M0581, M0582, M0583, M0584, M0585, M0586, M0587, M0588, M0589, M059, M0590, M0591, M0592, M0593, M0594, M0595, M0596, M0597, M0598, M0599, M06, M060, M0600, M0601, M0602, M0603, M0604, M0605, M0606, M0607, M0608, M0609, M061, M0610, M0611, M0612, M0613, M0614, M0615, M0616, M0617, M0618, M0619, M062, M0620, M0621, M0622, M0623, M0624, M0625, M0626, M0627, M0628, M0629, M063, M0630, M0631, M0632, M0633, M0634, M0635, M0636, M0637, M0638, M0639, M064, M0640, M0641, M0642, M0643, M0644, M0645, M0646, M0647, M0648, M0649, M068, M0680, M0681, M0682, M0683, M0684, M0685, M0686, M0687, M0688, M0689, M069, M0690, M0691, M0692, M0693, M0694, M0695, M0696, M0697, M0698, M0699, M315, M32, M320, M321, M328, M329, M33, M330, M331, M332, M339, M34, M340, M341, M342, M348, M349, M351, M353, M360 |
| **Ulcer disease** | 1 | K25, K250, K251, K252, K253, K254, K255, K256, K257, K259, K26, K260, K261, K262, K263, K264, K265, K266, K267, K269, K27, K270, K271, K272, K273, K274, K275, K276, K277, K279, K28, K280, K281, K282, K283, K284, K285, K286, K287, K289 |
| **Mild liver disease** | 1 | B18, B180, B181, B182, B188, B189, K70, K700, K701, K702, K703, K704, K709, K713, K714, K715, K717, K73, K730, K731, K732, K738, K739, K74, K740, K741, K742, K743, K744, K745, K746, K760, K762, K763, K764, K768, K769, Z944 |
| **Diabetes** | 1 | E100, E101, E106, E108, E109, E110, E111, E116, E118, E119, E120, E121, E126, E128, E129, E130, E131, E136, E138, E139, E140, E141, E146, E148, E149 |
| **Hemiplegia** | 2 | G041, G114, G801, G802, G81, G810, G8100, G8101, G8108, G811, G819, G82, G820, G821, G822, G823, G824, G825, G830, G831, G832, G833, G834, G839 |
| **Moderate or severe renal disease** | 2 | I120, I131, N032, N033, N0330, N0339, N034, N035, N036, N037, N052, N053, N054, N055, N056, N057, N18, N180, N181, N182, N183, N184, N185, N188, N189, N19, N250, Z490, Z491, Z492, Z940, Z992, Z992+0, Z992+1, Z992+8 |
| **Diabetes with end organ damage** | 2 | E102, E103, E104, E105, E107, E112, E1120, E1128, E113, E1130, E1138, E114, E1140, E1148, E115, E117, E122, E123, E124, E125, E127, E132, E133, E134, E135, E137, E142, E143, E144, E145, E147 |
| **Cancer** | 2 | C00, C000, C001, C002, C003, C004, C005, C006, C008, C009, C01, C02, C020, C021, C022, C023, C024, C028, C029, C03, C030, C031, C039, C04, C040, C041, C048, C049, C05, C050, C051, C052, C058, C059, C06, C060, C061, C062, C068, C069, C07, C08, C080, C081, C088, C089, C09, C090, C091, C098, C099, C10, C100, C101, C102, C103, C104, C108, C109, C11, C110, C111, C112, C113, C118, C119, C12, C13, C130, C131, C132, C138, C139, C14, C140, C142, C148, C15, C150, C151, C152, C153, C154, C155, C158, C159, C16, C160, C161, C162, C163, C164, C165, C166, C168, C169, C169+0, C169+8, C17, C170, C171, C172, C173, C178, C179, C18, C180, C181, C182, C183, C184, C185, C186, C187, C188, C189, C189+0, C189+8, C19, C20, C21, C210, C211, C212, C218, C22, C220, C221, C222, C223, C224, C227, C229, C23, C24, C240, C241, C248, C249, C25, C250, C251, C252, C253, C254, C254+0, C254+8, C257, C258, C259, C259+0, C259+8, C26, C260, C261, C268, C269, C30, C300, C301, C31, C310, C311, C312, C313, C318, C319, C32, C320, C321, C322, C323, C328, C329, C33, C34, C340, C341, C342, C343, C348, C349, C37, C38, C380, C381, C382, C383, C384, C388, C39, C390, C398, C399, C40, C400, C401, C402, C403, C408, C409, C41, C410, C411, C412, C413, C414, C418, C419, C43, C430, C431, C432, C433, C434, C435, C436, C437, C438, C439, C45, C450, C451, C452, C457, C459, C46, C460, C461, C462, C463, C467, C4670, C4671, C4672, C4678, C468, C469, C47, C470, C471, C472, C473, C474, C475, C476, C478, C479, C48, C480, C481, C482, C488, C49, C490, C491, C492, C493, C4930, C4938, C494, C4940, C4948, C495, C4950, C4958, C496, C498, C499, C50, C500, C501, C502, C503, C504, C505, C506, C508, C509, C51, C510, C511, C512, C518, C519, C52, C53, C530, C531, C538, C539, C54, C540, C541, C542, C543, C548, C549, C55, C56, C57, C570, C571, C572, C573, C574, C577, C578, C579, C58, C60, C600, C601, C602, C608, C609, C61, C62, C620, C621, C629, C63, C630, C631, C632, C637, C638, C639, C64, C65, C66, C67, C670, C671, C672, C673, C674, C675, C676, C677, C678, C679, C68, C680, C681, C688, C689, C69, C690, C691, C692, C693, C694, C695, C696, C698, C699, C70, C700, C701, C709, C71, C710, C711, C712, C713, C714, C715, C716, C717, C718, C719, C72, C720, C721, C722, C723, C724, C725, C728, C729, C73, C74, C740, C741, C749, C75, C750, C751, C752, C753, C754, C755, C758, C759, C76, C760, C761, C762, C763, C764, C765, C767, C768, C81, C810, C811, C812, C813, C814, C817, C819, C82, C820, C821, C822, C823, C824, C825, C826, C827, C829, C83, C830, C831, C832, C833, C834, C835, C836, C837, C838, C839, C84, C840, C841, C842, C843, C844, C845, C846, C847, C848, C849, C85, C850, C851, C852, C857, C859, C88, C880, C881, C882, C883, C884, C887, C889, C90, C900, C901, C902, C903, C91, C910, C911, C912, C913, C914, C915, C916, C917, C918, C919, C92, C920, C921, C922, C923, C924, C925, C926, C927, C928, C929, C93, C930, C931, C932, C933, C937, C939, C94, C940, C941, C942, C943, C944, C945, C946, C947, C95, C950, C951, C952, C957, C959, C96, C960, C961, C962, C963, C964, C965, C966, C967, C968, C969, C97 |
| **Moderate or severe liver disease** | 3 | I850, I859, I864, I982, K704, K711, K721, K729, K765, K766, K767 |
| **Metastatic cancer** | 6 | C77, C770, C771, C772, C773, C774, C775, C778, C779, C78, C780, C781, C782, C783, C784, C785, C786, C787, C788, C79, C790, C791, C792, C793, C794, C795, C796, C797, C798, C799, C80, C80+0, C800, C809 |
| **AIDS** | 6 | B20, B200, B201, B202, B203, B204, B205, B206, B207, B208, B209, B21, B210, B211, B212, B213, B217, B218, B21, B22, B220, B221, B222, B227, B24, B24+0, B24+1, B24+9, Z21 |
