# Supplementary material for: Impact of interhospital competition on mortality of patients operated on for colorectal cancer faced to hospital volume and rurality: A cross-sectional study
Source: PLoS One. 2024 Jan 25;19(1):e0291672. doi: 10.1371/journal.pone.0291672 (PMC10810549; doi:10.1371/journal.pone.0291672)
Supplement: S2 Appendix — (DOCX) [file pone.0291672.s002.docx]

**APPENDIX 2***: THE CODES OF COMMON CLASSIFICATION OF MEDICAL ACTS: 11TH VERSION*

| ***THE ACT*** | ***COMMON CLASSIFICATION OF MEDICAL ACTS CODES*** |
| --- | --- |
| **Left colectomy:** | HHFA002, HHFA006, HHFA010, HHFA014, HHFA017, HHFA024, HHFC040, HHFA003. |
| **Right colectomy** | HHFA008, HHFA009, HHFA026, HHFC296 |
| **Recto-sigmoid resection:** | HJFA001, HJFA002, HJFA004, HJFA011, HJFC031 |
| **Rectal resection:** | HJFA006, HJFA012, HJFA017, HJFC023, HJFA005, HJFA007, HJFA019 |
| **Total colectomy** | HHFA004, HHFA005, HHFA021, HHFA022, HHFA028, HHFA029, HHFA030, HHFA031, HHFA003 |
| **Transverse colectomy** | HHFA018, HHFA023 |
| **Local removal of colorectal cancer** | HHFC001, HJFA003, HJFA010, HJFA013, HJFA014, HJFA018 |
| **Multiple resections** | an association of two or more to type of procedures. |

* **Total colectomy**, **transverse colectomy** and **local removal of colorectal cancer** are grouped into **other procedures.**
