# Supplementary material for: Impact of interhospital competition on mortality of patients operated on for colorectal cancer faced to hospital volume and rurality: A cross-sectional study
Source: PLoS One. 2024 Jan 25;19(1):e0291672. doi: 10.1371/journal.pone.0291672 (PMC10810549; doi:10.1371/journal.pone.0291672)
Supplement: S3 Appendix — (DOCX) [file pone.0291672.s003.docx]

**APPENDIX 1***: THE CODES OF INTERNATIONAL CLASSIFICATION OF DISEASE: 10^TH^ VERSION*

| ***THE DIAGNOSIS*** | ***ICD-10 CODES*** |
| --- | --- |
| **Obesity** | E66 |
| **Malnutrition** | E43, E44, E46 |
| **Neo-adjuvant treatment:** | Z511 and Z510 |
