# Supplementary material for: Impact of interhospital competition on mortality of patients operated on for colorectal cancer faced to hospital volume and rurality: A cross-sectional study
Source: PLoS One. 2024 Jan 25;19(1):e0291672. doi: 10.1371/journal.pone.0291672 (PMC10810549; doi:10.1371/journal.pone.0291672)
Supplement: Attachment — Submitted filename: revision.docx [file pone.0291672.s004.docx]

I congratulate the authors for the work made on a large number of patients.

The very accurate results are interesting, as they show the correlation between mortality rate after colon-rectal cancer and the increasing hospital competition, independently of hospital volume and rurality.

On the other hand, the absence of control for surgeons and hospital quality, limits the study, as these two parameters could influence the mortality.

The study is well written in English.

I do not have any further recommendation.
