# Supplementary material for: Impact of interhospital competition on mortality of patients operated on for colorectal cancer faced to hospital volume and rurality: A cross-sectional study
Source: PLoS One. 2024 Jan 25;19(1):e0291672. doi: 10.1371/journal.pone.0291672 (PMC10810549; doi:10.1371/journal.pone.0291672)
Supplement: Attachment — Submitted filename: response to reviewer.docx [file pone.0291672.s005.docx]

**Response to the editorial board.**

**1. Please ensure that your manuscript meets PLOS ONE's style requirements, including those for file naming. The PLOS ONE style templates can be found at** 
[**https://journals.plos.org/plosone/s/file?id=wjVg/PLOSOne_formatting_sample_main_body.pdf**](https://journals.plos.org/plosone/s/file?id=wjVg/PLOSOne_formatting_sample_main_body.pdf) **and** 
[**https://journals.plos.org/plosone/s/file?id=ba62/PLOSOne_formatting_sample_title_authors_affiliations.pdf**](https://journals.plos.org/plosone/s/file?id=ba62/PLOSOne_formatting_sample_title_authors_affiliations.pdf)

*We revise the manuscript according to the two documents.*

**2. Thank you for submitting the above manuscript to PLOS ONE. During our internal evaluation of the manuscript, we found significant text overlap between your submission and previous work in the Methods.
We would like to make you aware that copying extracts from previous publications, especially outside the methods section, word-for-word is unacceptable. In addition, the reproduction of text from published reports has implications for the copyright that may apply to the publications.**

**Please revise the manuscript to rephrase the duplicated text, cite your sources, and provide details as to how the current manuscript advances on previous work. Please note that further consideration is dependent on the submission of a manuscript that addresses these concerns about the overlap in text with published work.
We will carefully review your manuscript upon resubmission and further consideration of the manuscript is dependent on the text overlap being addressed in full. Please ensure that your revision is thorough as failure to address the concerns to our satisfaction may result in your submission not being considered further.**

*We understand the remark of the editor concerning the method section. We modify the section describing the database.*

*Data were extracted from a National Health Database (the “Programme de Médicalisation des Systèmes d’Informations”, PMSI), which is a mandatory billing system for all hospitalization in France, either for public or private hospital (19). The aim of the PMSI is to set the budget of hospitals based on hospital activity. The external validity of this database has been tested with other national registry (20, 21, 22).*

*Nevertheless, the overlap highlighted by the publisher is due to previous publications by the same authors using the same database.*

**3. You indicated that ethical approval was not necessary for your study. We understand that the framework for ethical oversight requirements for studies of this type may differ depending on the setting and we would appreciate some further clarification regarding your research. Could you please provide further details on why your study is exempt from the need for approval and confirmation from your institutional review board or research ethics committee (e.g., in the form of a letter or email correspondence) that ethics review was not necessary for this study? Please include a copy of *the correspondence as an "Other" file.***

*In the PMSI database, each patient is assigned a unique identifier, which remains unchanged over time. Thus, linkage between different hospital stays in different hospitals is possible. Since the identifier is anonymous, patient consent was not required. This study complied with MR005 ("'Méthodologies de Référence") and the access to the database was submitted for authorization by the National Commission on Informatics and Liberty (CNIL, authorization n° 01947391).*

*We modify the text :*

*Access to the database was requested from and granted by the National Commission on Informatics and Liberty (“Commission Nationale de l'Informatique et des Libertés”, CNIL). As the identifier is anonymous, patient consent was not required. This study complied with MR005 ("'Méthodologies de Référence") and the access to the database was submitted for authorization by the National Commission on Informatics and Liberty (CNIL, authorization n° 01947391)*

**4. In your Data Availability statement, you have not specified where the minimal data set underlying the results described in your manuscript can be found. PLOS defines a study's minimal data set as the underlying data used to reach the conclusions drawn in the manuscript and any additional data required to replicate the reported study findings in their entirety. All PLOS journals require that the minimal data set be made fully available. For more information about our data policy, please see** [**http://journals.plos.org/plosone/s/data-availability**](http://journals.plos.org/plosone/s/data-availability)**.**

**"Upon re-submitting your revised manuscript, please upload your study’s minimal underlying data set as either Supporting Information files or to a stable, public repository and include the relevant URLs, DOIs, or accession numbers within your revised cover letter. For a list of acceptable repositories, please see** [**http://journals.plos.org/plosone/s/data-availability#loc-recommended-repositories**](http://journals.plos.org/plosone/s/data-availability#loc-recommended-repositories)**. Any potentially identifying patient information must be fully anonymized.**

**Important: If there are ethical or legal restrictions to sharing your data publicly, please explain these restrictions in detail. Please see our guidelines for more information on what we consider unacceptable restrictions to publicly sharing data:** [**http://journals.plos.org/plosone/s/data-availability#loc-unacceptable-data-access-restrictions**](http://journals.plos.org/plosone/s/data-availability#loc-unacceptable-data-access-restrictions)**. Note that it is not acceptable for the authors to be the sole named individuals responsible for ensuring data access.**

**We will update your Data Availability statement to reflect the information you provide in your cover letter.**

*As previous publication in PLOS ONE using the same database (*[*https://doi.org/10.1371/journal.pone.0260483*](https://doi.org/10.1371/journal.pone.0260483)*), the access to the data is restricted related to sensitive datas with high risk of rei-dentification of patients (city, date of hospitalization, identification of center).*

*Here, you can find a part of the document of law associated to the database access.*

*“I undertake to take all necessary precautions to preserve the security of the information contained in the PMSI files, and in particular to prevent it from being distorted, damaged or communicated to unauthorized third parties.*

*I undertake not to retrocede or divulge to third parties the information contained in the PMSI files in any form whatsoever.*

*I undertake not to carry out any reconciliation, interconnection, linking or matching with any file containing directly or indirectly nominative data or any information likely to reveal the identity of a person and/or his/her state of health.”*

*You can contact the health data hub at :*

[*https://www.health-data-hub.fr/contact*](https://www.health-data-hub.fr/contact)*.*

**5. Please include your full ethics statement in the ‘Methods’ section of your manuscript file. In your statement, please include the full name of the IRB or ethics committee who approved or waived your study, as well as whether or not you obtained informed written or verbal consent. If consent was waived for your study, please include this information in your statement as well.**

*As we state before at the point Nb3 :*

*Access to the database was requested from and granted by the National Commission on Informatics and Liberty (“Commission Nationale de l'Informatique et des Libertés”, CNIL). As the identifier is anonymous, patient consent was not required. This study complied with MR005 ("'Méthodologies de Référence") and the access to the database was submitted for authorization by the National Commission on Informatics and Liberty (CNIL, authorization n° 01947391)*

**6. We note that Figures 1 and 2 in your submission contain map images which may be copyrighted. All PLOS content is published under the Creative Commons Attribution License (CC BY 4.0), which means that the manuscript, images, and Supporting Information files will be freely available online, and any third party is permitted to access, download, copy, distribute, and use these materials in any way, even commercially, with proper attribution. For these reasons, we cannot publish previously copyrighted maps or satellite images created using proprietary data, such as Google software (Google Maps, Street View, and Earth). For more information, see our copyright guidelines:** [**http://journals.plos.org/plosone/s/licenses-and-copyright**](http://journals.plos.org/plosone/s/licenses-and-copyright)**.
 
We require you to either (1) present written permission from the copyright holder to publish these figures specifically under the CC BY 4.0 license, or (2) remove the figures from your submission:
 
a. You may seek permission from the original copyright holder of Figures 1 and 2 to publish the content specifically under the CC BY 4.0 license.  
 
We recommend that you contact the original copyright holder with the Content Permission Form (**[**http://journals.plos.org/plosone/s/file?id=7c09/content-permission-form.pdf**](http://journals.plos.org/plosone/s/file?id=7c09/content-permission-form.pdf)**) and the following text:
“I request permission for the open-access journal PLOS ONE to publish XXX under the Creative Commons Attribution License (CCAL) CC BY 4.0 (**[**http://creativecommons.org/licenses/by/4.0/**](http://creativecommons.org/licenses/by/4.0/)**). Please be aware that this license allows unrestricted use and distribution, even commercially, by third parties. Please reply and provide explicit written permission to publish XXX under a CC BY license and complete the attached form.”
 
Please upload the completed Content Permission Form or other proof of granted permissions as an "Other" file with your submission.
 
In the figure caption of the copyrighted figure, please include the following text: “Reprinted from [ref] under a CC BY license, with permission from [name of publisher], original copyright [original copyright year].”
 
b. If you are unable to obtain permission from the original copyright holder to publish these figures under the CC BY 4.0 license or if the copyright holder’s requirements are incompatible with the CC BY 4.0 license, please either i) remove the figure or ii) supply a replacement figure that complies with the CC BY 4.0 license. Please check copyright information on all replacement figures and update the figure caption with source information. If applicable, please specify in the figure caption text when a figure is similar but not identical to the original image and is therefore for illustrative purposes only.
The following resources for replacing copyrighted map figures may be helpful:
 
USGS National Map Viewer (public domain):** [**http://viewer.nationalmap.gov/viewer/**](http://viewer.nationalmap.gov/viewer/) **The Gateway to Astronaut Photography of Earth (public domain):** [**http://eol.jsc.nasa.gov/sseop/clickmap/**](http://eol.jsc.nasa.gov/sseop/clickmap/) **Maps at the CIA (public domain):** [**https://www.cia.gov/library/publications/the-world-factbook/index.html**](https://www.cia.gov/library/publications/the-world-factbook/index.html) **and** [**https://www.cia.gov/library/publications/cia-maps-publications/index.html**](https://www.cia.gov/library/publications/cia-maps-publications/index.html) **NASA Earth Observatory (public domain):** [**http://earthobservatory.nasa.gov/**](http://earthobservatory.nasa.gov/) **Landsat:** [**http://landsat.visibleearth.nasa.gov/**](http://landsat.visibleearth.nasa.gov/) **USGS EROS (Earth Resources Observatory and Science (EROS) Center) (public domain):** [**http://eros.usgs.gov/#**](http://eros.usgs.gov/) **Natural Earth (public domain):** [**http://www.naturalearthdata.com/**](http://www.naturalearthdata.com/)

*Figures were drawn with Leaflet package on R using OpenStreetMap covered by Open data commons. We add the use of the package and the reference on figure legends.*

**7. Please include captions for your Supporting Information files at the end of your manuscript, and update any in-text citations to match accordingly. Please see our Supporting Information guidelines for more information:** [**http://journals.plos.org/plosone/s/supporting-information**](http://journals.plos.org/plosone/s/supporting-information)**.**

*Modifications have been done in the methods section:*

*Charlson comorbidity index (the version of Quan and colleagues) was calculated based on the patient's overall diagnosis, using the International Classification of Diseases,10th version (ICD-10), in the last two years prior to surgery (APPENDIX 1) (24, 25). The surgical procedures were defined using the CCAM (APPENDIX 2). The history of obesity, neo-adjuvant treatment and malnutrition were defined using also ICD-10 codes (APPENDIX 3).*

*And at the end of manuscript*

**APPENDIX 1.** *CHARLSON COMORBITY SCORE CALCULATION*

***APPENDIX 2****: THE CODES OF COMMON CLASSIFICATION OF MEDICAL ACTS: 11TH VERSION*

***APPENDIX 3****: THE CODES OF INTERNATIONAL CLASSIFICATION OF DISEASE: 10^TH^ VERSION*

**8. Please review your reference list to ensure that it is complete and correct. If you have cited papers that have been retracted, please include the rationale for doing so in the manuscript text, or remove these references and replace them with relevant current references. Any changes to the reference list should be mentioned in the rebuttal letter that accompanies your revised manuscript. If you need to cite a retracted article, indicate the article’s retracted status in the References list and also include a citation and full reference for the retraction notice.**

*Reference list has been updated.*

**Response to reviewer**

**Reviewer #1**

**I congratulate the authors for the work made on a large number of patients.**

**The very accurate results are interesting, as they show the correlation between mortality rate after colon-rectal cancer and the increasing hospital competition, independently of hospital volume and rurality.**

*We thank reviewer 1 for this kind comment*.

**On the other hand, the absence of control for surgeons and hospital quality, limits the study, as these two parameters could influence the mortality.**

*We agree with reviewer 1. It could be very interesting to adjust the analysis on surgeon activity volume and control quality of surgeon, and as well add some marker on hospital quality. The database does not contain these datas, we cannot access to the surgeon volume activity nor the rate of completion of text book outcome or the rate of guidelines application for example as we discuss in the limits in the study.*

**The study is well written in English.**

**I do not have any further recommendation.**

*We thanks reviewer 1 for this comment*.

**Reviewer #2:**

**There are some typos and grammar errors, i.e., in the title says: cross-sectionnal study instead of “a cross-sectional study”. In the discussion section also says:
. These findings are consistent with a study from previous studies.
. The activity per year per center in non-competitive hospital was higher than in highly competitive center and the surgical activity of the center impact 30-day mortality as previously describe by several authors.
These sentences sound a little weird. I suggest editing help from someone with full professional proficiency in English.**

*We thanks the reviewer 2 for these helpful comments, we modify the text according the reviewer comments.*

*The title :*

*IMPACT OF INTERHOSPITAL COMPETITION ON MORTALITY OF PATIENTS OPERATED ON FOR COLORECTAL CANCER FACED TO HOSPITAL VOLUME AND RURALITY: A CROSS-SECTIONAL STUDY*

*In the discussion :*

*These findings are consistent with previous studies (28-30).*

*Indeed, the activity volume in a non-competitive hospital may be higher than in a highly competitive center. As surgical activity is a major factor associated to the post-operative mortality, it is very important to take this into account (28-30).*

**Reference style should be reviewed. In some cases, references are written after the dot, instead of before, i.e,
From 2004 to 2008, pro-competition reforms were gradually introduced.(8)
However, other studies show that competition increases mortality.(13, 14)**

*We thank the reviewer 2, we have modified the references style according to the comment.* **Data are not available because there are some restrictions on publicly sharing data. Those are specified (Data were collected from a National Health Database)**

*This is true, we can only provide data in acceptable demand according to the ethical French from the CNIL (Commission nationale de l'informatique et des libertés : national commitee of informatic law and liberties)* **The article is interesting and methodologically correct. Statistical analysis also seems the most appropriate for the variables they handled and the objectives of the study. The use of the national database to obtain data from both private and public hospitals allows the sample to be large in order to draw conclusions.**

*We thank the reviewer 2 for this comment.* **I have some doubts relating to the conclusions. It is indicated that increasing hospital competition decreases the 30-day mortality rate after colorectal cancer surgery independently of the hospital volume and the rurality of the hospital. However, they previously indicate that mortality was higher in hospitals with higher volumes. Besides, the author refers to Amrani's study, which also found an association with hospital volume.**

**The table also indicates statistical significance of the volume of the hospital:
Hospital’s caseload/year
OR [CI] p.value OR [CI] p.value
> 100.74 (0.63, 0.86) <0.001 0.69 (0.59, 0.80) <0.001**

**Instead, hospital status does seem to have had no influence.**

*We agree with the reviewer comment, hospital volume is major factor of post operative death.*

*We modify the discussion of this section. We corrected the impact of volume activity on mortality: high volume activity is correlated to lower mortality as your tables show it :*

*We also found a significant effect of hospital caseload, patients’ characteristics and age on mortality. These findings are consistent with previous studies, Amrani and al. found an association between lower 90-day post-operative mortality and higher volume activity.*

*We state that competition is independent factor associated to the mortality, regarding hospital volume and rurality, because after including all these variables in the regression model, there is still a significant association between competition and mortality. We agree, hospital volume had a very significant impact on postoperative mortality. We modify the conclusion about it :*

*The results of our studies show that increasing hospital competition independently decreases the 30-day mortality rate after colorectal cancer surgery. Hospital caseload, patients’ characteristics and age also impact the post-operative mortality.*

**I am also left with the question of knowing the differences between the 2 periods studied (before and after the reform) by the Gobillon study that they refer to.**

*After re-reading this sentence, it adds nothing to the discussion : we decide to remove “Another aspect of Gobillon's study is that it compares the impact of competition on mortality between two periods, the period before the pro-competitive reform and the period after this reform.” from the discussion.*
